# Graves’ disease-induced immune thrombocytopenic purpura in an African female: a case report

**DOI:** 10.1186/s13256-023-03869-2

**Published:** 2023-04-05

**Authors:** Kwabena Oteng Agyapong, Aba A. Folson, Kate Fiador, Cecilia Kootin-Sanwu, Martha Tufuor, Sally Afua Bampoh, Henrietta Fiscian, Roland Wonkyi

**Affiliations:** 1 Department of Internal Medicine, Greater Accra Regional Hospital (Ridge Hospital), Ridge, P. O. Box GP 473, Accra, Ghana; 2 Department of Hematology, Greater Accra Regional Hospital (Ridge Hospital), Ridge, P. O. Box GP 473, Accra, Ghana; 3 School of Medicine, University of Health and Allied Sciences, PMB 31, Ho, Volta Region Ghana

**Keywords:** Idiopathic thrombocytopenic purpura, ITP, Platelet, Antithyroid, Glucocorticoid

## Abstract

**Background:**

Immune thrombocytopenic purpura is a condition associated with an unusual, unexplained, and sometimes very severe reduction in the level of platelets in the blood. Though documented, its association with Graves’ disease is not very common and can easily be missed or misdiagnosed, leading to excessive bleeding and mortality. Treatment with steroids and antithyroid medications has been shown to be beneficial in correcting thrombocytopenia in these patients, although the response is varied. We report on a patient with Graves’ disease who presents with immune thrombocytopenic purpura.

**Case presentation:**

A 37-year-old Ghanaian female presented to our hospital’s emergency department with a complaint of palpitations, difficulty breathing, easy fatigue, and headaches. She had been referred from a peripheral hospital as a case of thrombocytopenia, severe anemia, and anterior neck swelling. She was diagnosed with Graves’ disease 2 years ago, became euthyroid during treatment, but defaulted. On further examination and investigation, she was diagnosed with immune thrombocytopenic purpura and was also found to have elevated free T3 and T4, and suppressed thyroid stimulating hormone. She also had high thyroid autoantibodies. She was initially started on oral prednisolone but there was no stabilization of platelets until methimazole was introduced, which improved and normalized her platelet count.

**Conclusion:**

The association of Graves’ disease with immune thrombocytopenic purpura, though documented, is uncommon, and very few cases have been reported thus far. There have not been any reported cases in Ghana or Sub-Saharan Africa and hence, clinicians should be aware of this association when investigating immune thrombocytopenic purpura and should consider Graves’ disease as a possible cause. From this study, we observed that there was no improvement in platelet count following the use of corticosteroid therapy until methimazole was started.

## Introduction

Immune thrombocytopenic purpura (ITP) has been associated with autoimmune diseases such as systemic lupus erythematosus (SLE), antiphospholipid syndrome, and infections with hepatitis C infection, *Helicobacter pylori* infection, and severe acute respiratory syndrome coronavirus 2 (SARS-CoV-2) [1]. Graves’ disease, being autoimmune in etiology, is no exception, even though there is a paucity of data in this area.

Clinically, Graves’ disease is characterized by a diffusely enlarged thyroid gland with associated symptoms of thyroid hormone overactivity, including palpitations, heat intolerance, diarrhea, and oligomenorrhea [2]. Additionally, there is evidence of platelet-related autoantibody production in some patients with Graves’ disease [3], specifically CD4 T cell-mediated signaling of B cells to produce autoantibodies against platelet membrane glycoprotein (GP) 11b/111a [4]. Other *in vitro* studies suggest mechanisms including megakaryocyte suppression by autoantibodies [5]. The diagnosis of ITP, especially in association with other conditions, requires a high index of suspicion considering the lack of specific diagnostic tests. Subsequent management involves the use of glucocorticoids and antithyroid medications, with varying responses to the platelet count.

Our case reports the likely association between Graves’ disease and ITP, and the potential exacerbating effects of high thyroid hormone levels and high titers of circulating autoantibodies on platelet count.

## Case presentation

A 37-year-old Ghanaian female presented to the emergency department of our hospital with a complaint of palpitations, difficulty breathing, easy fatigue, and headaches. She had been referred from a peripheral hospital as a case of thrombocytopenia, severe anemia, and anterior neck swelling.

Relevant past medical history included a diagnosis of Graves’ disease 2 years before this admission, for which she was put on antithyroid medication. Her family and psychosocial history were not of note. She became euthyroid during treatment but subsequently defaulted on her medications and long-term follow-up. Four months before her current admission, she noticed heavy menstrual bleeding with progressive weight loss. Four days before the presentation, the patient experienced dizziness, easy fatigue, profuse sweating, and tremors. At the referring hospital where she was initially admitted, she was transfused with whole blood after laboratory results showed anemia with hemoglobin (Hb) of 3.2 g/dl (normal: 11.5–16.5 g/dl) with mean corpuscular volume (MCV) of 79 fl (normal: 76–99 fl), white blood cell (WBC) count of 8.3 × 10^9^/l (normal: 4–10 × 10^9^/l) and platelet (PLT) count of 1 × 10^9^/l (normal: 150–450 × 10^9^/l). Physical examination showed bruising and ecchymosis all over her body and petechiae on her lower lips with gum bleeding. She had an anterior neck swelling that moved on swallowing, was diffuse, nontender, measured 3 × 3 cm, and with no thyroid bruit. There was exophthalmos demonstrated by the Naffziger sign. She also had a blood-soaked diaper with significant amounts of clots. All other examination systems were unremarkable.

During admission, her initial blood pressure was 121/68 mmHg. She had a heart rate of 175 beats per minute with a weak and thready pulse, her respiratory rate was 40 cycles per minute, and peripheral oxygen saturation of 97% on oxygen via face mask at a flow rate of 6 liter/minute. On primary assessment, she was in severe respiratory distress and restless, severely pale, and had cold extremities with an axillary temperature of 36.5 °C. She was also noted to be bleeding per vaginum. Based on her laboratory results from the referral center (Hb: 3.2 g/dl and PLT: 1 × 10^9^/l), she was immediately scheduled to receive fresh frozen plasma and packed cells. She, however, suffered a cardiac arrest during the initial assessment at the emergency. Cardiopulmonary resuscitation (CPR) was commenced and return of spontaneous circulation (ROSC) was achieved. She was subsequently sent to the high-dependency unit (HDU) after resuscitation for further assessment and management.

A repeat complete blood count (CBC) showed a Hb of 5.4 g/dl, MCV of 82.5 fl, WBC count of 6.56 × 10^9^/l, and PLT of 4.0 × 10^9^/l. On admission day 2, her posttransfusion peripheral film comment indicated two populations of cells: microcytic hypochromic and normocytic normochromic red blood cells. Additionally, neutrophilia and markedly reduced platelets without clumping were seen. These findings were suggestive of multiple blood transfusions and probable autoimmune disease, and a diagnosis of ITP was considered. She was initially started on oral prednisolone 60 mg daily for 5 days, with a plan to gradually taper over a period of 4 weeks and supported with parenteral esomeprazole 40 mg 12 hourly, and tranexamic acid 1 g 8 hourly for 48 hours, while awaiting other laboratory test results. Hepatitis B and C virus and human immunodeficiency virus (HIV) serology were negative. Thyroid function testing revealed a low thyroid stimulating hormone (TSH) of 0.006 IU/ml (normal: 0.38–5.33 IU/ml), and elevated serum-free T3 of 9.2 pmol/l (normal: 3.5–7.8 pmol/l) and serum-free T4 of 42.1 pmol/l (7.9–18.5 pmol/l). The thyroid autoantibodies demonstrated elevated thyroperoxidase antibodies (TPOAb) levels of 58.91 IU/ml (normal: < 30 IU/ml), TSH receptor autoantibodies (TRAb) levels of 14.91 IU/l (normal: < 1.80 IU/l), and elevated thyroglobulin antibodies (TgAb) levels of 32.51 IU/ml (normal; < 4.11 IU/ml). An ultrasound of the thyroid revealed heterogeneous and hyperechoic glands of average size, with a mild increase in vascularity but no nodularity. A diagnosis of Graves’ disease-induced ITP was made, and she was commenced on oral propranolol 40 mg twice daily and oral methimazole 20 mg daily. The patient was followed-up at the endocrine and hematology clinics. Three weeks following admission, her thyroid function tests, Hb, and platelet results were all within normal limits. After 5 months of treatment and monitoring, TSH receptor autoantibodies were requested, and they were within normal limits (TRAb 0.05 IU/l).

A summary of the patient’s CBC results from admission to her most recent review is presented in Table 1.

**Table 1:** Summary of patient’s complete blood count results from admission to most recent review

CBC parameter	Date (dd/mm) from admission to the last review								
	*17/01	18/01	21/01	22/01	25/01	**29/01	31/01	03/02	05/02
Hb (g/dl)	3.2	5.4	3.0	4.6	4.3	6.6	7.2	9.0	12.5
WBC (× 10^9^/l)	7.85	6.56	5.0	28.6	8.3	11.7	12.7	5.7	6.0
Platelet (× 10^9^/l)	1.0	4.0	7.0	10.0	6.0	5.0	27	88	235

*CBC* complete blood count, *Hb* hemoglobin, *WBC* white blood cell

*Date of starting oral prednisolone

**Date oral prednisolone was stopped and oral methimazole started

## Discussion

As many as 80% of cases of hyperthyroidism are due to Graves’ disease, with the overall prevalence of hyperthyroidism in the USA being 1.2% and the incidence being 20 per 100,000 to 50 per 100,000 of the population. Graves’ disease is more common in females between the ages of 20 and 50 years [7]. Manifestations of ophthalmopathy, which vary in severity and have a course that is typically independent of the thyroid disease, may include proptosis, periorbital edema, exposure keratitis, extraocular muscle infiltration, lid lag, and lid retraction. A thyroid function test reveals a suppressed TSH and elevated free T3 (FT3) and free T4 (FT4) [6]. Our patient presented with exophthalmos and an enlarged thyroid gland, with both FT3 and FT4 levels elevated above the normal reference range. A diagnosis of Graves’ disease can also be made with antibody titers to the thyroid gland. About 95% of patients have antibodies to thyroid peroxidase (TPO) and about 50% to thyroglobulin (TG). Antibodies to the TSH receptor strongly support Graves’ disease [7]. In the case report presented, the diagnosis of Graves’ disease was made by the presence of elevated levels of autoantibodies.

Immune thrombocytopenic purpura is an acquired hematological disorder caused by the autoimmune destruction of platelets with values below 100 × 10^9^/l in the absence of other causes of thrombocytopenia, such as viral infections, rheumatic diseases, or drugs. An investigation for other causes of thrombocytopenia, such as HIV, Hepatitis B and C viruses, drugs, or rheumatologic diseases, such as SLE and antiphospholipid syndrome, is therefore imperative before making this diagnosis [8]. ITP can vary from being asymptomatic to causing severe, life-threatening bleeding. In severe cases, treatment options include platelet transfusion, intravenous immunoglobulin (IVIG), and glucocorticoids, with the main aim of maintaining platelets at a level to prevent spontaneous bleeding [9].

The association between thyroid disease pharmacotherapy and ITP is controversial. Several case studies report a complete reversal of thrombocytopenia after the use of antithyroid medications, whereas others report no improvement in platelet count after achieving euthyroidism [8]. There have been case reports of ITP induced by Graves’ disease where patients were treated with IVIG or oral prednisolone but with little or no improvement until carbimazole was started [10]. Our patient was started on prednisolone, but there was no improvement in platelet levels until methimazole was started, which normalized the platelet count and the Hb levels.

From this case report, we observed that there was no improvement in platelet count following the use of corticosteroid therapy until a thionamide was started. In all cases of ITP with Graves’ disease, we suggest thyroid function and autoantibody tests are conducted and appropriate treatment commenced, especially in the setting of poor response to standard ITP treatment protocols.

## Conclusion

In Sub-Saharan African countries such as Ghana, with limited healthcare settings, a case of an association between Graves’ disease and ITP is rarely diagnosed, partly due to under-diagnosis or late referrals to centers where a multidisciplinary team can institute care. To the best of our knowledge, no such case study from this region has been reported in the literature yet. There are several treatment approaches and subsequently, variable outcomes when treating ITP with underlying Graves’ disease. It has been observed that glucocorticoids can sometimes reverse thrombocytopenia in some patients yet have no effect in others. In this case study, glucocorticoids did not reverse thrombocytopenia in our patient; however, reversal of thrombocytopenia and normalization of Hb count was noticed when antithyroid therapy was administered.

## Data Availability

All data generated or analyzed during this study are included in this published article (and its additional information files).

## Abbreviations

ITP: Immune thrombocytopenic purpura
SLE: Systemic lupus erythematosus
SARS-CoV-2: Severe acute respiratory syndrome coronavirus 2
GP: Glycoprotein
HB: Hemoglobin
WBC: White blood cell
MCV: Mean corpuscular volume
CPR: Cardiopulmonary resuscitation
ROSC: Return of spontaneous circulation
HDU: High-dependency unit
TSH: Thyroid stimulating hormone
TPOAb: Thyroperoxidase antibodies
TRAb: TSH receptor autoantibodies
TgAb: Thyroglobulin antibodies
FT3: Free T3
FT4: Free T4
IVIG: Intravenous immunoglobulin
